# Comparison of the flavor qualities between two varieties of *Mercenaria mercenaria*

**DOI:** 10.1038/s41598-023-39757-4

**Published:** 2023-08-11

**Authors:** Zhidong Zhang, Suhua Chen, Aihua Chen, Yanshun Xu, Yu Zhang, Wenwen Yu, Yi Cao, Chaofeng Jia, Yangping Wu

**Affiliations:** 1 Marine Fisheries Research Institute of Jiangsu Province, 31 Jiaoyu Road, Chongchuan District, Nantong, 226007 Jiangsu China; 2 Jiangsu Marine Economic Shellfish Research and Development Center, Lvsi Town, Qidong, Nantong, 226241 Jiangsu China; 3 State Key Laboratory of Food Science and Technology, School of Food Science and Technology, Jiangnan University, 1800 Lihu Avenue, Binhu District, Wuxi, 214122 Jiangsu China

**Keywords:** Biochemistry, Metabolomics

## Abstract

The saltwater hard clam *Mercenaria mercenaria* (*M. mercenaria*) as a representative of low-value shellfish, enhancing its flavor quality, is the key to enter the high-end market. Nevertheless, there has not been reported research on the flavor quality of *M. mercenaria*. This study compared the flavor quality of selective and non-selective saltwater hard clams of *M. mercenaria* by using various indicators: proximate component, free amino acids, nucleotides, and metabolomic analysis. The results indicated that selective breeding contributed to the significant improvement contents of crude protein, flavor-associated free amino acids (glutamic acid, aspartic acid, proline, etc.), and nucleotides (AMP) (*P* < 0.05). Then, the metabolome was utilized to assess the metabolite changes in the pre/post-selective breeding of *M. mercenaria* and further understand the flavor characteristics and metabolic status. In the metabolomics assay, among the 3143 quantified metabolites, a total of 102 peaks were identified as significantly different metabolites (SDMs) between the selective and non-selective varieties of *M. mercenaria* (VIP > 1 and *P* < 0.05). These results can provide new insights for future research on improving the quality of saltwater bivalves through selective breeding.

## Introduction

Bivalves are popular seafood in markets all over the world because of their abundant bioactive compounds and unique pleasant-tasting flavor^1,2^. The saltwater hard clam *Mercenaria mercenaria* (*M. mercenaria*), native to the east coast of the United States and Canada, is one of the economically significant bivalve mollusks^3,4^. In 1997, it was imported by academician Fu-sui Zhang from the United States to China and became a vital aquatic product in China's coastal areas^5,6^. Because of its strong adaptability to the environment, even living for several days exposed to the air, its aquaculture yield is very high, and they can be well sold alive after harvest or during transportation^5,7,8^. Meanwhile, according to consumers, it tastes somewhat inferior to other clams, such as *Meretrix meretrix*, *Cyclina sinensis*, and *Ruditapes philippinarum*, resulting in a deficient market price. With the accelerated advancement of aquaculture and resident living standards, consumers are paying growing attention to seafood quality, and consumption has become more quality-oriented than price-oriented^9^. Consequently, as a representative of low-value shellfish, enhancing its flavor quality is the key to entering the high-end market.

Flavor substances and components are representative indexes to assess the quality of seafood embodied in their rich proteins and amino acids^9,10^. Amino acids are flavor precursors that can alter taste indirectly via the Melanoidin reaction^11^. Additionally, nucleotides also can affect the flavor of flesh through interaction with G protein coupled receptors in tongue epithelial cells^12^. As we all known, the flavor of aquatic animals' flesh is influenced by both interior (such as parental genotype, body tissue, development period, etc.) and exterior factors (such as dietary composition, survival environment, etc.)^10,13^. Accordingly, at present, there are three ways to enhance the flesh quality and taste of aquatic animals. The first is to add exogenous nutrients such as dietary carbohydrates, protein, and lipids to enhance their own nutrition^14–16^. The second is to upgrade farming models so that breeding animals have an appropriate living environment that is conducive to the accumulation of their own nutrients^17–19^. Lastly, the nutritional characteristics of aquatic animals are strengthened by gene editing, crossbreeding, or selective breeding^10,20,21^.

As a classical approach, mass selection is widely used in selective breeding programs for aquatic animals, which can be easily implemented by establishing several selected generations to assess the breeding potential of desired traits^22,23^. Previously, mass selective breeding programs for most aquatic animals offered the possibility of exerting high selection pressure to improve growth traits, and their genetic diversity could be efficiently maintained through genetic mating strategies^23–27^. Selective breeding has been demonstrated in oysters and scallops, an intrinsic means of improving edible flesh quality^20,23,28^. But to date, information on the flesh quality of *M. mercenaria* has been limited.

Hence, this study was conducted to compare the amino acids and nucleotide components and metabolic characteristics in the pre/post-selective breeding of *M. mercenaria* via liquid chromatography mass spectrometry (LC–MS), which will provide new insights for future research on improving the flavor quality of saltwater bivalves through selective breeding.

## Materials and methodology

### Sample preparation and collection

**Figure 1:**
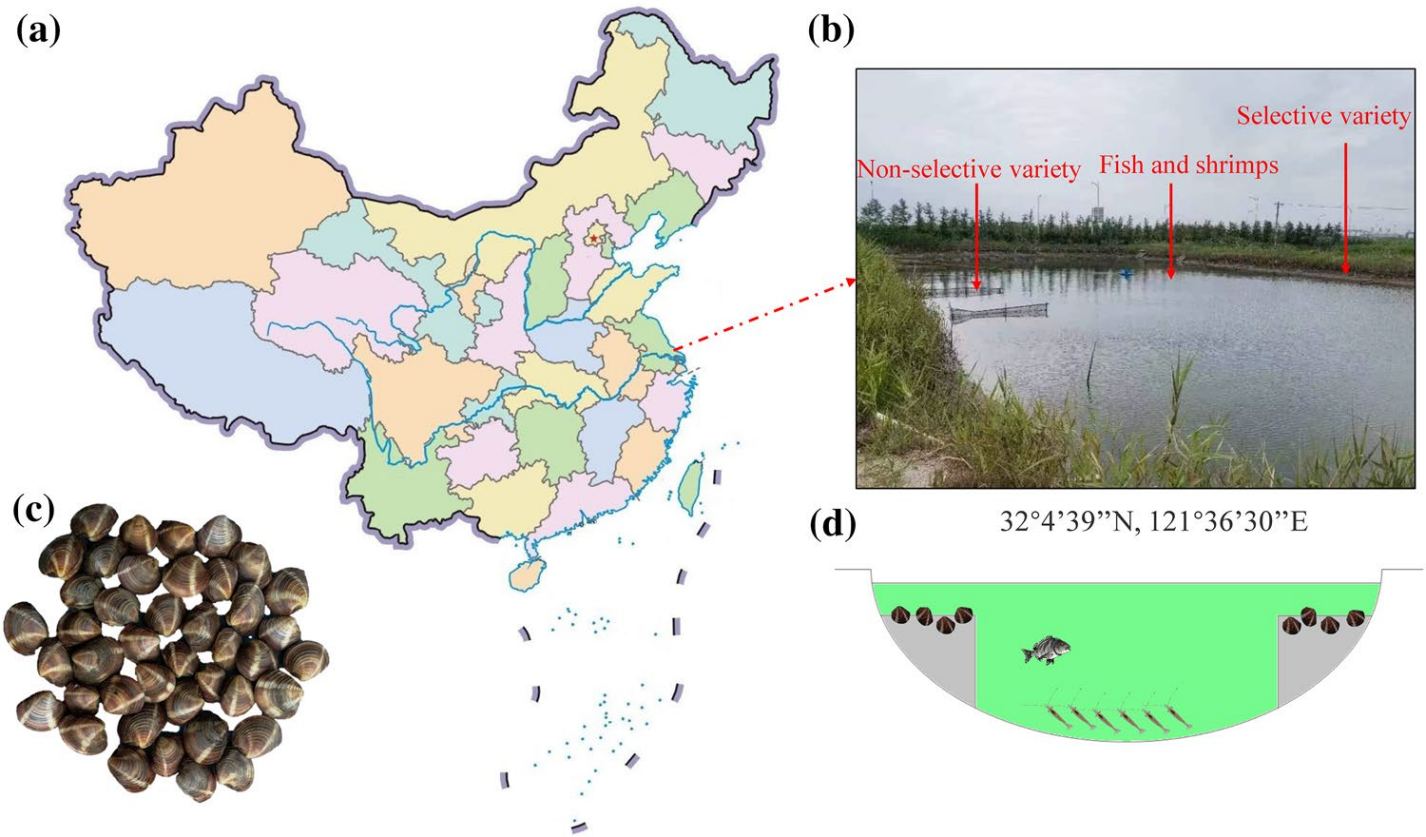
(**a**) Location of the field area in Jiangsu province, China (coordinates: N 32°4ʹ 39″, E 121°36ʹ30″), (**b**) landscape of the studied ecological farming pond, (**c**) photographs of saltwater hard clam (*Mercenaria mercenaria*) for the selective variety, (**d**) model diagram of the polyculture pond.

The saltwater hard clam *M. mercenaria*, including the non-selective variety and selective variety, were obtained from the Jiangsu Marine Economic Shellfish R & D center. The selective variety of *M. mercenaria* was selected continuously though gray shell color and grain weight (two-generation selection) (Fig. 1c). Specifically, *M. mercenaria* was selected breeding in 2017 and the breeding base group (first filial generation) was constructed in 2019 with the 5% selection proportion. The second filial generation of the *M. mercenaria* population was obtained in 2021 with a 1% selection proportion. The non-selective variety was not the selected population. Two varieties were bred and reared at the same time in the same pond (coordinates: N 32°4ʹ39″, E 121°36ʹ30″) (Fig. 1a, b). The polyculture pond structure (Fig.1c, d) and rearing method were referred to Zhang by adding several black porgy (*Acanthopagrus schlegelii*)^29^. Two varieties of *M. mercenaria* (body weight of the selective variety = 7.68 ± 1.71 g, body weight of the non-selective variety = 7.26 ± 2.19 g) were placed in the sand-filtered seawater for 48 h for purification. The edible flesh of two varieties was anatomized and collected on ice, and three individuals were mixed into a sample at random of each variety. After washing with 0.01 mol phosphate buffered saline, each sample was instantly covered with liquid nitrogen and kept at −80 °C to analyze the approximate composition, free amino acids, 5-nucleotides, and metabolome.

### Flavor indicator detection

**Figure 2:**
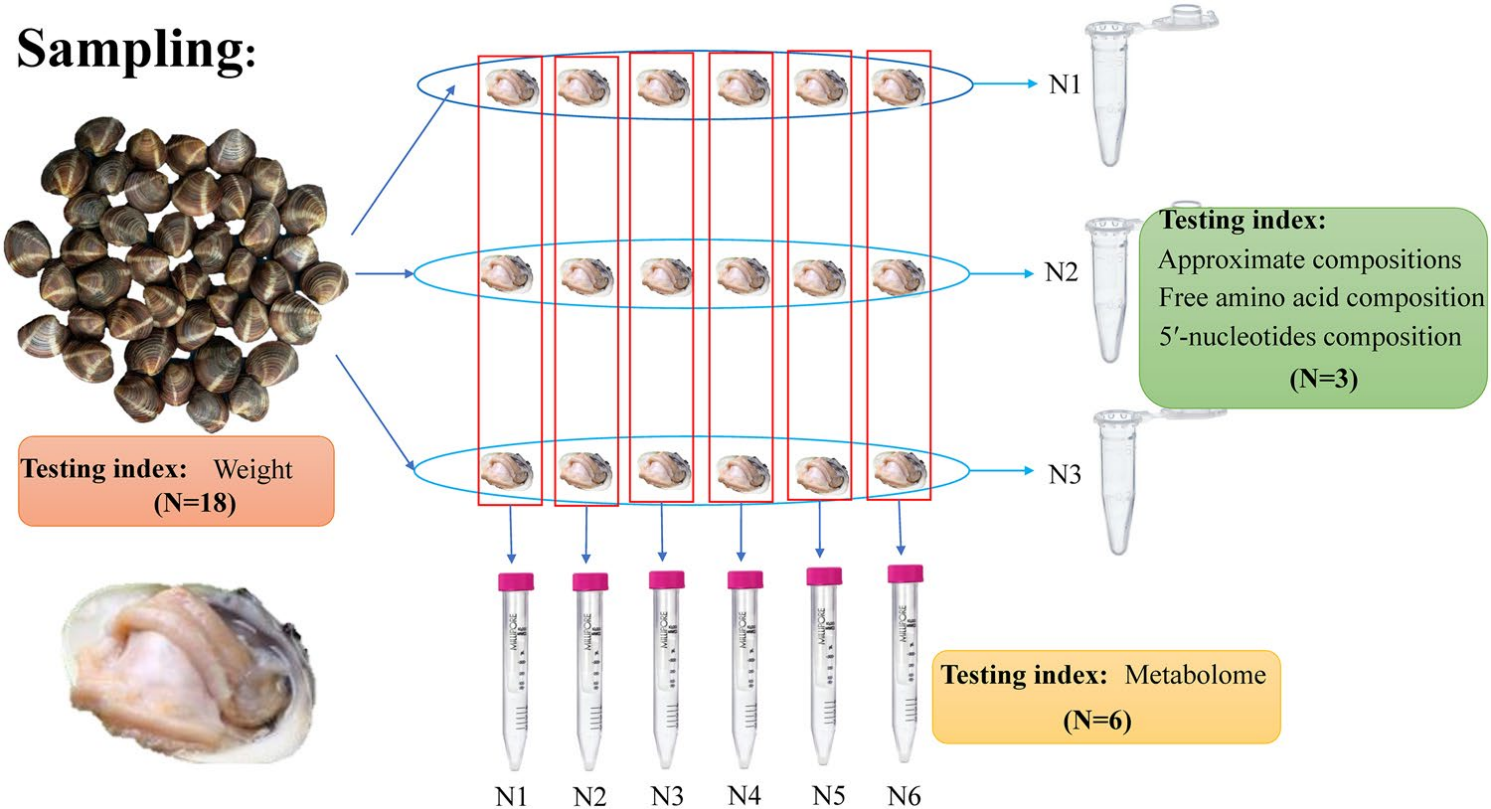
Sampling methods and number of sample.

The analysis methods of approximate composition, free amino acid, and 5ʹ-nucleotide assay referred to the method published by the previous studies^30^. Twelve samples were prepared for LC–MS, referring to the method of Sangster et al. and Want et al.^31,32^. The method of Wu et al. was used for metabolite extraction and LC–MS/MS detection^33^. See Fig. 2 for specific sampling methods.

### Data analysis

The MS raw data (.wiff) files were converted to the mzXML format using ProteoWizard and processed using the R package XCMS^33,34^. The process includes peak deconvolution, alignment, and integration. The detected metabolites in two varieties of clams were enriched and analyzed based on the Kyoto Encyclopedia of Genes and Genomes database (KEGG) (http://www.genome.jp/kegg/), and the KEGG advanced bubble map was drawn according to the FDR (*P*-value correction) detected by metabolome^35^. Data were expressed as the mean value ± standard deviation (mean ± SD). The differences were compared using *Student’s t test* by SPSS software (version 19.0).

## Results

### Proximate edible flesh composition of *M. mercenaria* between the selective variety and non-selective variety

The approximate compositions of the two varieties of *M. mercenaria* are listed in Table 1. *M. mercenaria* had the highest compositional value of moisture, followed by crude protein and ash, and the lowest compositional value of crude fat. There were no significant differences in moisture, ash, and crude fat between the selective variety and non-selective variety (*P* > 0.05). However, the content of crude protein in the selective variety (9.54 ± 0.10%) was significantly higher than that in the non-selective variety (7.97 ± 0.45%, *P* < 0.05).

**Table 1:** Comparison of proximate compositions between two varieties of *Mercenaria mercenaria* (%, based on wet weight).

Parameter	Non-selective variety	Selective variety
Moisture	85.71 ± 1.24	85.25 ± 1.08
Ash	2.50 ± 0.22	2.54 ± 0.08
Crude fat	0.83 ± 0.13	0.83 ± 0.09
Crude protein	7.97 ± 0.77	9.54 ± 0.18*

Data are expressed as mean ± SD. (n = 3). The asterisk (*) indicates a significant difference from the Student’s *t* test in the same row at *P* < 0.05.

### Free amino acid composition of *M. mercenaria* between the selective variety and non-selective variety

As listed in Table 2, the most abundant amino acids in the edible flesh of *M. mercenaria* were glutamic acid (Glu), glycine (Gly), and arginine (Arg) and the contents of cysteine (Cys) and serine (Ser) were very low. The contents of Glu, Gly, phenylalanine (Phe), proline (Pro), and total amino acids (TAAs) in *M. mercenaria* were significantly increased (*P* < 0.05), while the contents of Arg, alanine (Ala), tyrosine (Tyr) were significantly decreased (*P* < 0.05) after two-generation selective breeding. Furthermore, the contents of flavor amino acids, including sweet amino acid (sweet AA), salt amino acid (salt AA), sour amino acid (sour AA), umami amino acid (umami AA) were significantly increased (*P* < 0.01), while there was no significant difference in the content of bitter amino acids (bitter AA) after two-generation selective breeding (*P* = 0.05).

**Table 2:** Comparison of free amino acid compositions between two varieties of *Mercenaria mercenaria* (mg/100 g, based on wet weight).

Free amino acids	Non-selective variety	Selective variety
Aspartic acid	220.61 ± 25.19	254.96 ± 7.88
Glutamic acid	530.68 ± 27.41	709.77 ± 17.18**
Serine	8.60 ± 0.89	13.82 ± 3.66
Histidine	40.16 ± 3.75	40.79 ± 4.67
Glycine	371.26 ± 14.19	643.95 ± 18.10**
Threonine	78.03 ± 6.26	78.50 ± 3.82
Arginine	536.62 ± 2.24	429.60 ± 19.48**
Alanine	549.75 ± 12.95	457.76 ± 10.36**
Tyrosine	66.80 ± 1.47	54.53 ± 0.80**
Cysteine	8.54 ± 0.92	8.31 ± 0.71
Valine	73.74 ± 3.21	67.81 ± 4.77
Methionine	49.82 ± 6.54	43.47 ± 5.23
Phenylalanine	44.79 ± 4.47	76.80 ± 4.65**
Isoleucine	46.51 ± 2.18	50.44 ± 2.07
Leucine	67.67 ± 2.08	71.73 ± 6.49
Lysine	128.61 ± 4.06	131.04 ± 2.25
Proline	56.54 ± 3.27	66.27 ± 2.60*
Sweet AA	1192.78 ± 16.14	1391.33 ± 1.86**
Salt AA	751.29 ± 6.11	964.73 ± 12.04**
Bitter AA	398.11 ± 5.31	419.39 ± 12.13
Sour AA	791.45 ± 5.73	1005.52 ± 10.44**
Umami AA	1672.30 ± 14.74	2066.43 ± 13.65**
TAAs	2878.75 ± 22.26	3199.54 ± 20.63**

Data are expressed as mean ± SD (n = 3). The asterisks (*) and (**) indicate a significant difference from the Student’s *t* test in the same row at *P* < 0.05 and *P* < 0.01, respectively. Sweet AA: the sum of Gly, Ala, Ser, Thr, Lys and Pro. Salt AA: the sum of Asp and Glu. Bitter AA: the sum of Met, Val, Leu, Ile, Phe, Ser, Tyr and His. Sour AA: the sum of Asp, Glu and His. Umami AA: the sum of Asp, Glu, Gly and Ala.

*TAA* total amino acids.

### 5ʹ-nucleotides composition of *M. mercenaria* between the selective variety and non-selective variety

The 5ʹ-nucleotides compositions of *M. mercenaria* were listed in Table 3. The content of adenylic acid (AMP) of the selective variety (176.41 ± 7.16 mg/100 g) was higher than that of the non-selective variety (152.98 ± 6.78 mg/100 g, *P* < 0.05). The content of guanylic acid (GMP) of the selective variety (19.98 ± 2.83 mg/100 g) was higher than that of the non-selective variety (33.95 ± 5.98 mg/100 g, *P* < 0.05). However, there were no significant differences in the content of inosinic acid (IMP) between the selective variety (26.43 ± 2.81 mg/100 g) and the non-selective variety (25.27 ± 3.92 mg/100 g, *P* > 0.05).

**Table 3:** Comparison of 5ʹ-nucleotides compositions between two varieties of *Mercenaria mercenaria* (mg/100 g, based on wet weight).

Nucleotides	Non-selective variety	Selective variety
AMP	152.98 ± 6.78	176.41 ± 7.16*
GMP	33.95 ± 5.98	19.98 ± 2.83*
IMP	25.27 ± 3.92	26.43 ± 2.81

Data are expressed as mean ± SD (n = 3). The asterisk (*) indicates a significant difference from the Student’s *t* test in the same row at *P* < 0.05.

### Multivariate statistical analysis of the metabolite patterns of *M. mercenaria* between the selective variety and non-selective variety

**Figure 3:**
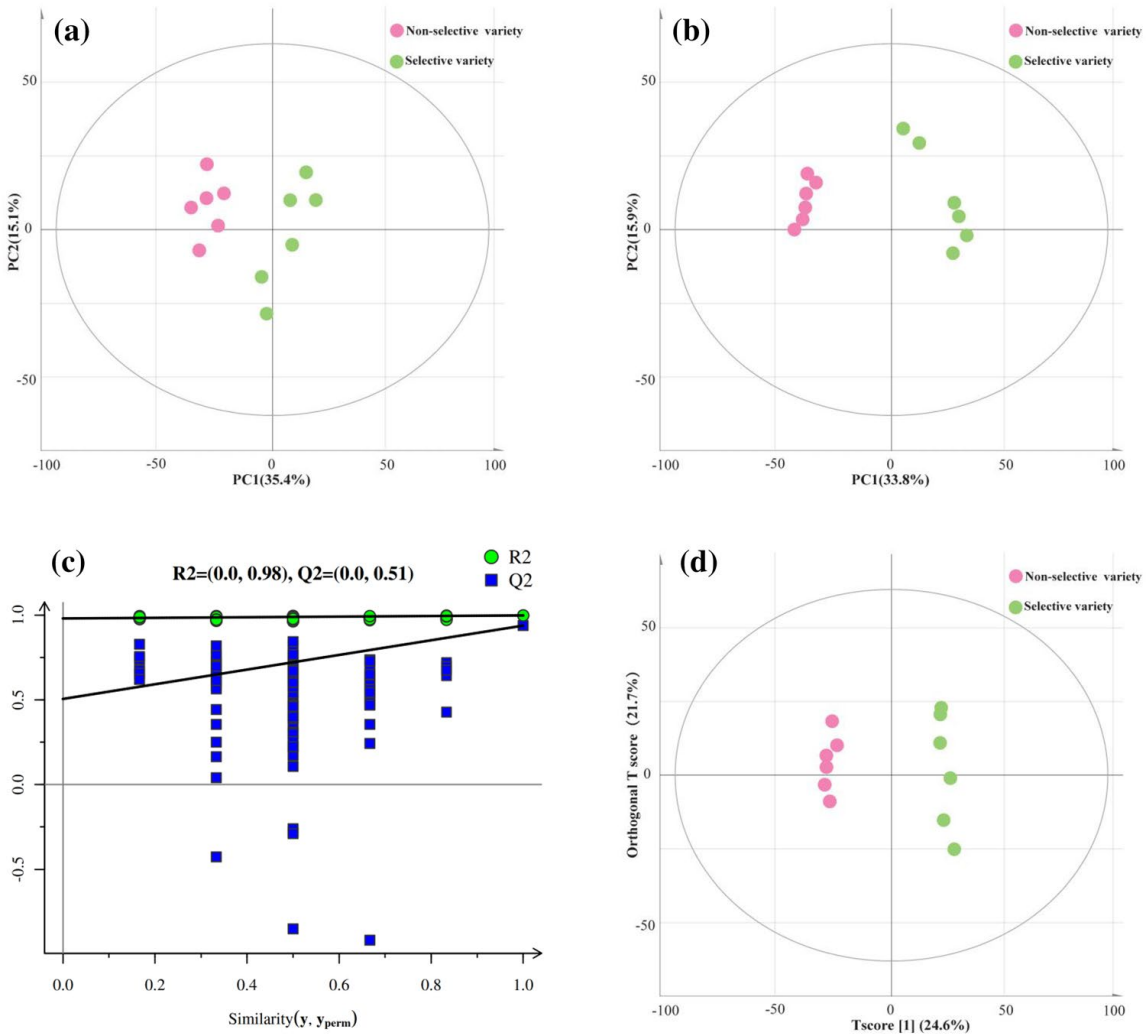
multivariate statistical analysis and PLS-DA permutation test of *M. mercenaria* between the selective variety and non-selective variety. (**a**) PCA score plot, (**b**) PLS-DA score plot, (**c**) PLS-DA permutation test plot; the green line represents the regression line for R2 and the blue line for Q2. (**d**) OPLS-DA score plot.

All edible flesh samples were subjected to multivariate statistical analysis methods such as principal component analysis (PCA), partial least squares discriminate analysis (PLS-DA), and orthogonal projection to latent structures discriminant analysis (OPLS-DA). All samples in the score plots were located inside the Hotelling's T-squared ellipse of 95% confidence in PCA, PLS-DA, and OPLS-DA, thereby indicating that no outlier was present among the analyzed samples (Figs. 2b, d and 3a). Specifically, as shown in the PCA (Fig. 3a), the samples of the non-selective variety and selective variety showed intragroup clustering and intergroup dispersion, indicating differences between the samples of the two varieties. Besides, the OPLS-DA score scatter plot showed that clear separation and discrimination were found between the pair-wise varieties and the R^2^X and R^2^Y values of the OPLS-DA model accounting for the variance were 0.560 and 0.997, respectively, which demonstrated that the OPLS-DA model can be applied to recognize the difference between two varieties of *M. mercenaria* (Fig. 3d). Permutation plots can be used as a standard to evaluate the reliability and effectiveness of the PLS-DA model and solve the problem of overfitting model. The reliability criteria are as follows: All blue Q2 points from left to right are lower than the original blue Q2 points at the right or the regression line of the points crosses with the abscissa or is less than 0. It can be seen that parameters considered for the classification were R^2^X = 0.565, R^2^Y = 0.997, and Q2 = 0.932 (Fig. 3b). The R2 (green circle) and Q2 (blue square) intercept values were 0.98 and 0.51 after 200 permutations. They as two parameters of the permutation test, represent model interpretability and model predictability, respectively (Fig. 3d). Our results suggest that the model has a good stable prediction and a low risk of overfitting (Fig. 3c).

### Significantly different metabolites (SDMs) of M. mercenaria between the selective variety and non-selective variety

**Figure 4:**
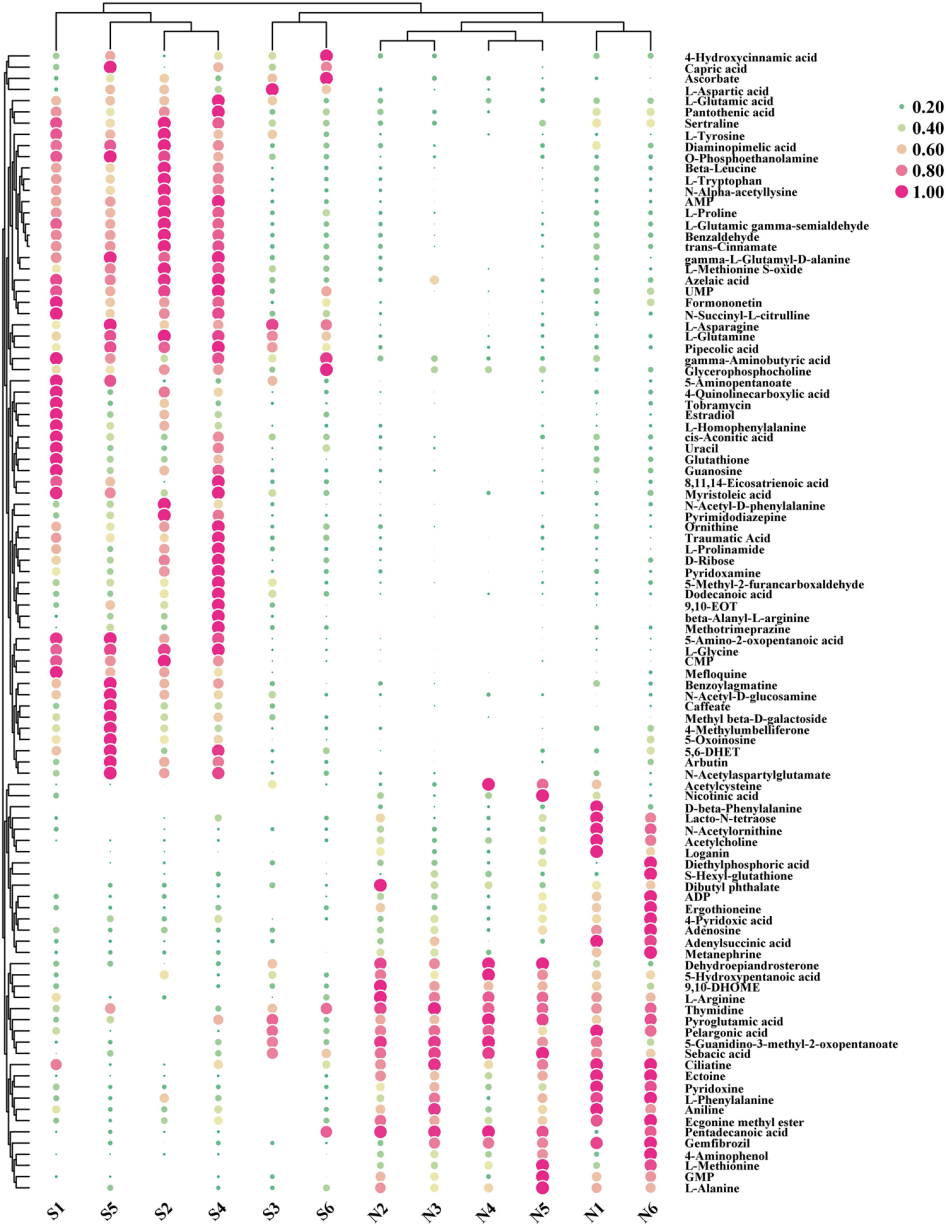
Agglomerate the hierarchical clustering heatmap of metabolites between the selective variety and non-selective variety. *S* selective variety, *N* non-selective variety.

Among the 3143 quantified metabolites, a total of 102 peaks were identified as SDMs between the selective and non-selective varieties of *M. mercenaria* (VIP > 1 and *P* < 0.05; Fig. 4). The metabolite distribution was visually split into upregulation and downregulation. Of the 102 SDMs, 65 metabolites were significantly upregulated in the selective variety compared to the non-selective variety, such as l-aspartic acid, l-glycine, l-proline, l-glutamic acid, l-glutamine, AMP, cytidylic acid (CMP), uridylic acid (UMP). By contrast, 37 metabolites in the selective variety were significantly downregulated compared to the non-selective variety. These metabolites mainly included L-arginine, L-phenylalanine, L-alanine, L-methionine, GMP (Fig. 4 and Table 4). These results are consistent with the free amino acid and 5ʹ-nucleotides composition described above.

**Table 4:** Identification nucleotides and amino acids of SDMs of *M. mercenaria* between the selective variety and non-selective variety.

Metabolites	Models	Mass	Formula	Mean N	Mean S	Foldchange	VIP	*P*-value
l-aspartic acid	[M + H]+	133.038	C_4_H_7_NO_4_	371,655,165.9	707,248,385.6	0.525	1.521	0.005
l-glycine	[M + H]+	155.070	C_2_H_5_NO_2_	23,243,321.67	248,210,483.1	0.094	1.408	0.005
l-proline	[M + H]+	115.063	C_5_H_9_NO_2_	5,590,355,308	17,648,882,532	0.317	1.521	0.008
l-tyrosine	[M + H]+	181.189	C_9_HNO_3_	531,490,310.245	2,073,714,044.258	0.256	1.668	0.005
l-glutamic acid	[M + H]+	147.053	C_5_H_9_NO_4_	3,627,110,974	4,747,567,292	0.764	1.430	0.031
l-alanine	[M + H]+	89.048	C_3_H_7_NO_2_	72,202,818.61	24,508,845.47	2.946	1.674	0.005
l-phenylalanine	[M + H]+	165.079	C_9_H_11_NO_2_	273,065,276.9	127,835,729	2.136	1.234	0.045
l-arginine	[M + H]+	174.112	C_6_H_14_N_4_O_2_	9,154,530,179	5,422,875,621	1.688	1.723	0.005
Beta-leucine	[M − H]−	131.095	C_6_H_13_NO_2_	541,817,875.6	1,787,889,156	0.303	1.335	0.045
AMP	[M + H]+	347.063	C_10_H_14_N_5_O_7_P	18,256,511.560	120,798,804.400	0.151	1.441	0.008
CMP	[M − H]−	323.052	C_9_H_14_N_3_O_8_P	602,615.132	13,202,853.980	21.909	1.420	0.008
UMP	[M + H]+	324.036	C_9_H_13_N_2_O_9_P	52,722,653.81	171,004,470.300	0.308	1.568	0.013
GMP	[M + H]+	363.058	C_10_H_14_N_5_O_8_P	220,797,460.6	81,841,349.2	2.698	1.604	0.005

[M + H]+ and [M − H]− represent the positive ion mode and negative ion mode, respectively. Mean N and Mean S represent the average of the non-selective and selective varieties (n = 6), respectively. Foldchange indicates the specific variable of the selective variety relative to the non-selective variety. VIP (variable importance in projection) describes the overall contribution of each variable to the model, and the threshold is usually set as VIP > 1.

### Characterization and functional analysis of key metabolic pathways

**Figure 5:**
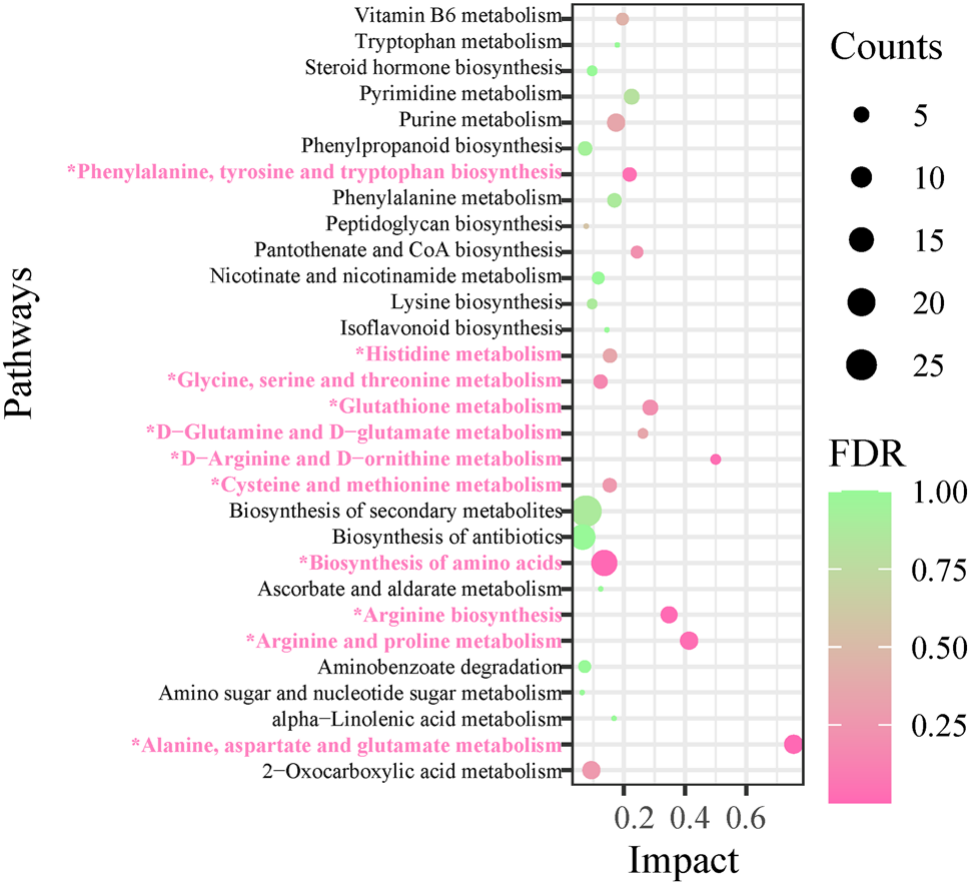
statistical scatter plot of pathway enrichment of significantly different metabolites. The x-axis and y-axis represented the enrichment impact values and metabolic pathways, respectively. The value of impact represents the ratio of significantly different metabolites to all metabolites annotated to this metabolic pathway; the larger the value of impact, the greater the degree of enrichment. FDR (*P*-value correction), ranging from 0 to 1, the closer to 0, the more significant the enrichment.

**Figure 6:**
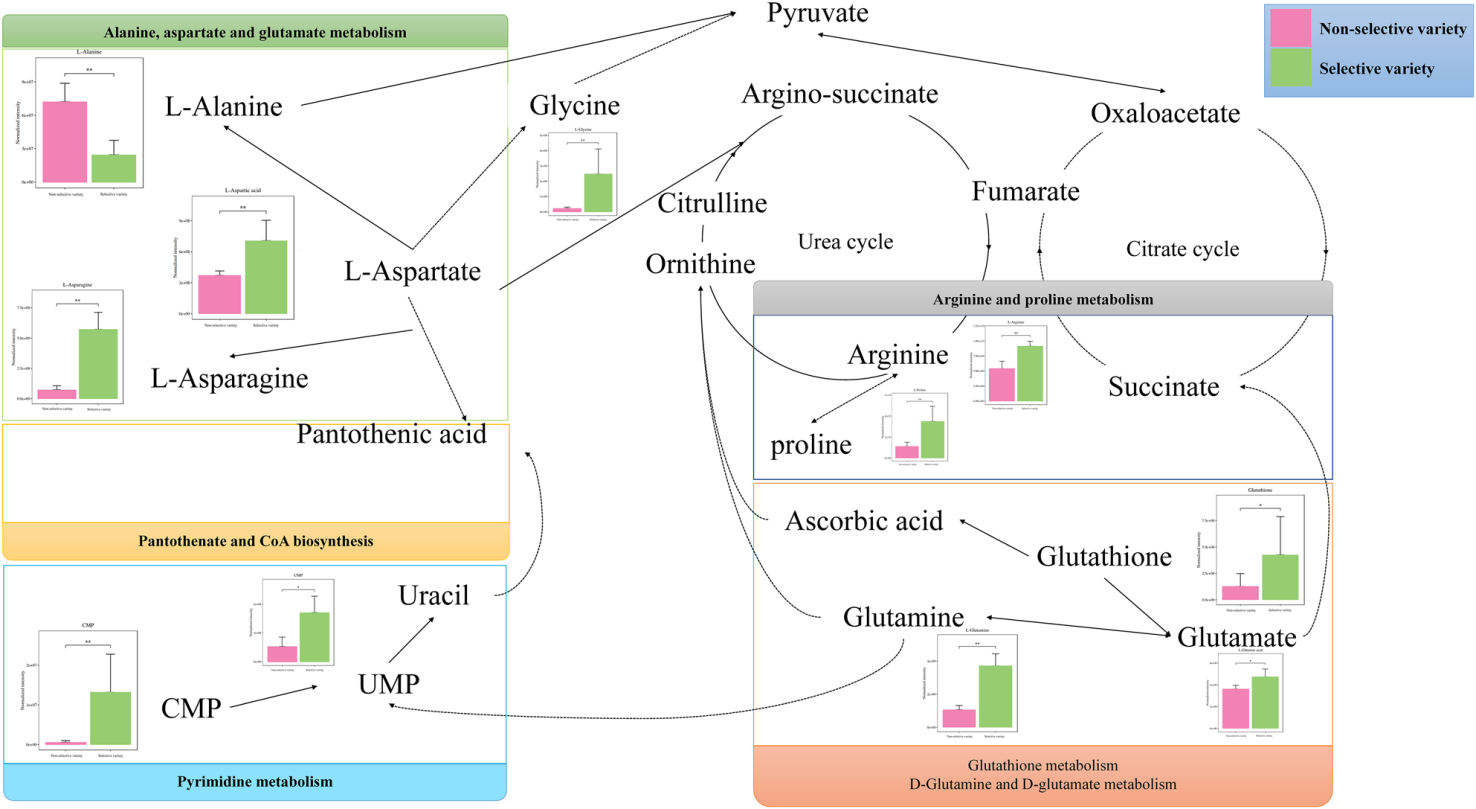
A schematic presentation of pathways indicated by altered metabolites after two generations selective breeding, according to Kyoto Encyclopedia of Genes and Genomes (http://www.genome.jp/kegg/). Asterisks (*) and (**) show significant differences between the non-selective and selective varieties at *P* < 0.05 and *P* < 0.01, respectively. Solid arrow: one-step biochemical reaction. Dashed arrow: indirect reaction.

In order to further understand the most relevant metabolic pathways to reveal the flavor metabolism of *M. mercenaria*, SDMs were imported into KEGG database for metabolic pathway analysis. A total of 73 pathways were obtained by comparing the non-selective and selective varieties and the top 30 pathways were presented in Fig. 4 (Supplementary Table 1 and Fig. 5). These pathways include alanine, aspartate and glutamate metabolism, arginine and proline metabolism, arginine biosynthesis, D-arginine and D-ornithine metabolism, glutathione metabolism, D-glutamine and D-glutamate metabolism, phenylalanine, tyrosine and cysteine and methionine metabolism and so on. Base on FDR (*P*-value correction) and impact values, alanine, aspartate and glutamate metabolism, arginine and proline metabolism, arginine biosynthesis, d-arginine and d-ornithine metabolism, glutathione metabolism, d-glutamine and d-glutamate metabolism were characterized as relevant pathways (Fig. 5). Thus, a key schematic overview was constructed to reveal the nutritional metabolism of *M. mercenaria* according to the reference diagrams stored in the KEGG database (Fig. 6).

## Discussion

The whole clams’ flesh was made into powder and tested for its approximate free amino acids, and 5ʹ-nucleotides composition, because everything except the shell is edible for bivalve mollusks. In the present study, the moisture content of* M. mercenaria* ranged from 84.28 to 86.45%, ash content ranged from 2.31 to 2.74%, and crude fat ranged from 0.72 to 0.98%, which is similar to its related species such as *Meretrix lusoria* and *M. meretrix*. However, crude protein content ranged from 7.34% to 9.50%, which is slightly lower than *M. lusoria* (9.09 -12.75%) and *M. meretrix* (9.22–10.90%)^30,36^. Moreover, for *M. mercenaria*, the crude protein content of the selective variety is significantly higher than that of the non-selective variety (*P* < 0.05), indicating that selective breeding of *M. mercenaria* can promote the bioaccumulation of crude protein.

Free amino acids are generally considered the key indicator for evaluating the flavor quality of seafood^37^. In the present study, seventeen amino acids were identified from two varieties of *M. mercenaria*, and the total free amino acids contents were 2878.75 ± 22.26 and 3199.54 ± 20.63 mg/100 g (dry weight), respectively. The most top five abundant free amino acids contributing to the taste in the two varieties of *M. mercenaria* were glutamic acid, glycine, arginine, alanine, and aspartic acid. It is known that free amino acids are the main contributors to taste, such as sweetness, salty, bitterness, sour, and umami^38^. For instance, Asp and Glu can take on a distinctive umami taste with natrium salt^39,40^. Gly and Ala can arouse the palate to produce a sense of sweetness and counterbalance bitterness and saltiness^41^. In the current study, the contents of Glu, Gly, Phe, and Pro were significantly increased (*P* < 0.05), while the contents of Arg, Ala, and Tyr were significantly decreased (*P* < 0.05). In general, the contents of sweet AA, salt AA, sour AA, umami AA were significantly increased (*P* < 0.01) after two-generation selective breeding. As an off-flavor amino acid, histidine has a distasteful flavor such as sourness or bitterness^10,30^. Fortunately, there were no significant differences in the content of bitter AA after two-generation selective breeding (*P* = 0.05).

5ʹ-nucleotides, mainly AMP, GMP and IMP, usually contribute to umami taste in food^42^. AMP has been confirmed as a taste-active component in seafood^7,30^. The GMP content is high in fungi, while the IMP content is high in fish and meat^7^. The umami taste is the result of the interaction of amino acids, nucleotides, and other substances. Studies have demonstrated that the combination of 5ʹ-nucleotides and MSG-like components (Glu and Asp) synergistically potentiated umami^30^. Likewise, the joint action of AMP and Imp can also strengthen the umami taste of food^30^. In the present study, the content of AMP was significantly increased after two-generation selective breeding of *M. mercenaria* (*P* < 0.05). These results indicated that *M. mercenaria* after two-generation selective breeding enhanced the pleasant taste compared to the non-selective variety. Interestingly, we selected shell color and growth performance as target traits instead of flavor, but the flavor of the selective variety was improved. We hypothesis that some genes that determine edible flesh flavor quality are linked to those that determine growth or shell color traits. Therefore, through selective breeding of growth traits and shell color for two generations, the flavor quality of offspring is indirectly enhanced and gradually accumulated. However, there were no significant differences in the content of Imp after two-generation selective breeding (*P* > 0.05), revealing that there is still room for further generations of selective breeding to improve the flavor quality of *M. mercenaria*.

Recently, LC–MS is a sensitive food taste analytical technique for determining flavor components and the corresponding metabolic profile characteristics^43^. PCA, PLS-DA, and OPLS-DA showed that samples of the non-selective variety and selective variety demonstrated intragroup clustering and intergroup dispersion, implying that there were obvious metabolic differences in the pre/post-selective breeding of *M. mercenaria*. In the current study, a schematic presentation of metabolic pathways was drawn, as indicated by altered metabolites after two generations selective breeding, referring to KEGG database^35^. These SDMs participated in alanine, aspartate and glutamate metabolism, arginine and proline metabolism, arginine biosynthesis, d-arginine and d-ornithine metabolism, glutathione metabolism, d-glutamine and d-glutamate metabolism, etc (Fig. 4). In the alanine, aspartate, and glutamate metabolism, glutathione metabolism, and d-glutamine, and d-glutamate metabolism, the contents of l-aspartate, l-asparagine, glutamine, glutamate, and glutathione in the selective variety were higher than those in the non-selective variety, which was consistent with juvenile pearl oyster *Pinctada maxima*^44^. This result confirmed that the selective variety had more umami flavor than the non-selective variety.

In addition to flavor, free amino acids have other biological functions in vivo. For example, aspartic acid participates in neurotransmission and protein biosynthesis ^16^. In this study, the content of Asp and crude protein increased 34.35 mg/100 g and 1.57% in the selective variety. This may be because the increase in aspartic acid promotes protein anabolism. In addition, glycine is the basic component of purines, creatine, glutathione, and other substances synthesized in the body, and widely exists in marine bivalves^16,45^. The glycine contents in the selective variety were higher than those in the non-selective variety. This result was consistent with the role of glycine as a common osmolyte in marine bivalves^44,46^. Previous studies revealed that glycine content was related to the health state of oysters and clams, with reductions in this amino acid occurring after infection with pathogens^47^, after exposure to heavy metals^48^, and under hypo-osmotic conditions^49^. Young et al. also demonstrated that glycine content is significantly reduced in poor-quality cohorts^50^. Thus, this study proposes that improved glycine content may reduce the occurrence of stress-induced disruptions in osmotic regulation, resulting in better growth performance of *M. mercenaria* in the selective variety compared to the non-selective variety. Glutathione and glutamic acid play vital roles in the defense against oxidative stress^16^. In the present study, the SDMs (glycine, glutathione, glutamic acid, etc.) were significantly enriched in these metabolic pathways, indicating that the antioxidant capacity of the selective variety was strengthened. There are studies showing obesity may be associated with altered amino acid metabolism^51^. On the basis of these results, we proposed that the contents of Gly increased significantly in the selective variety might alter arginine and proline metabolism, glutathione metabolism, d-glutamine and d-glutamate metabolism, subsequently leading to strengthen the antioxidant capacity of *M. mercenaria*, which then led to the difference in grain weight between selective variety and non-selective variety of *M. mercenaria*. In the meantime, the changes of amino acid metabolism status enhance the flavor quality of the selective varieties^16,44^. Because of the instability of genetic traits caused by two successive generations of breeding, we will continue to breed *M. mercenaria* for many years to come.

## Conclusion

In summary, selective breeding contributed to the significant improvement contents of crude protein, flavor-associated free amino acids (glutamic acid, aspartic acid, proline, etc.), and flavor-associated nucleotides (AMP) (*P* < 0.05). In the metabolomics assay, among the 3143 quantified metabolites, a total of 102 peaks were identified as SDMs between the selective and non-selective varieties of *M. mercenaria* (VIP > 1 and *P* < 0.05). These SDMs were significantly enriched in 73 pathways. These results confirmed that the selective variety had more umami flavor than the non-selective variety.

### Supplementary Information

Supplementary Table 1.

## Data Availability

The data used to support the findings of this study are available from the corresponding author upon request.

## References

[1] Grienke U, Silke J, Tasdemir D (2014). Bioactive compounds from marine mussels and their effects on human health. Food Chem..

[2] Ran Z, Zhang S, Zhu Y, Ke A, Xu J, Li Y, Liao K, Li S, Ran Y, Yan X (2018). Effect of salinity on volatiles in the razor clam investigated by head space-solid phase microextraction/gas chromatography–mass spectrometry. Fish. Sci..

[3] Hu Z, Song H, Zhou C, Yu ZL, Yang MJ, Zhang TD (2020). novo assembly transcriptome analysis reveals the preliminary molecular mechanism of pigmentation in juveniles of the hard clam *Mercenaria **mercenaria*. Genomics.

[4] Song, H., Guo, X. M., Sun, L. N., Wang, Q. H., Han, F. M., Wang, H. Y., Wray. G. A., Davidson, P., Wang, Q., Hu, Z., Zhou, C., Yu, Z. L., Yang, M. J., Feng, J., Shi, P., Zhou, Y., Zhang, L. B., & Zhang, T. The hard clam genome reveals massive expansion and diversification of inhibitors of apoptosis in Bivalvia. *BMC Biol*. **19**(1), 15 (2021). 10.1186/s12915-020-00943-9 PMC7831173 33487168

[5] Zhou C, Song H, Feng J, Hu Z, Yu ZL, Yang MJ, Shi P, Li YR, Guo YJ, Zhang T (2021). RNA-seq analysis and WGCNA reveal dynamic molecular responses to air exposure in the hard clam *Mercenaria **mercenaria*. Genomics.

[6] Zhou C, Song H, Yang MJ, Wang XC, Yu ZL, Hu Z, Shi P, Zhang T (2021). Single-molecule long-read (SMRT) transcriptome sequencing of *Mercenaria **mercenaria* reveals a powerful anti-apoptotic system critical for air exposure endurance. Comp. Biochem. Physiol. Part D Genomics Proteomics..

[7] Chiou TK, Lin JF, Shiau CY (1998). Changes in extractive components and glycogen in the edible meat of hard clam *Meretrix **lusoria* during storage at different temperatures. Fish. Sci..

[8] Hu Z, Feng J, Song H, Zhou CMJ, Wang SP, Yu ZL, Guo YJ, Li YR, Zhang T (2022). Metabolic response of *Mercenaria **mercenaria* under heat and hypoxia stress by widely targeted metabolomic approach. Sci. Total Environ..

[9] Jiang WD, Wen HL, Liu Y, Jiang J, Wu P, Zhao J, Feng L (2016). Enhanced muscle nutrient content and flesh quality, resulting from tryptophan, is associated with anti-oxidative damage referred to the Nrf2 and TOR signalling factors in young grass carp (*Ctenopharyngodon*
*idella*): Avoid tryptophan deficiency or excess. Food Chem..

[10] Cai L, Tong FL, Tang T, Ao ZP, Wei ZH, Yang FZ, Shu YQ, Liu SJ, Mai KS (2021). Comparative evaluation of nutritional value and flavor quality of muscle in triploid and diploid common carp: Application of genetic improvement in fish quality. Aquaculture.

[11] Bermúdez R, Franco D, Carballo J, Sentandreu MA, Lorenzo JM (2014). Influence of muscle type on the evolution of free amino acids and sarcoplasmic and myofibrillar proteins through the manufacturing process of celta dry-cured ham. Food Res. Int..

[12] Hoon MA, Adler E, Lindemeier J, Battey JF, Ryba NJ, Zuker CS (1999). Putative mammalian taste receptors: A class of taste-specific GPCRs with distinct topographic selectivity. Cell.

[13] Fuentes A, Ferńandez-Segovia I, Escriche I, Serra JA (2009). Comparison of physicochemical parameters and composition of mussels (*Mytilus*
*galloprovincialis* Lmk.) from different Spanish origins. Food Chem..

[14] Andersen SM, Assaad HI, Lin G, Wang JJ, Aksnes A, Wu GY, Espe M (2015). Metabolomic analysis of plasma and liver from surplus arginine fed Atlantic salmon. Front. Biosci. Elite.

[15] Ma QQ, Chen Q, Shen ZH, Li DL, Han T, Qin JG, Chen LQ, Du ZY (2017). The metabolomics responses of Chinese mitten-hand crab (*Eriocheir*
*sinensis*) to different dietary oils. Aquaculture.

[16] Yang CY, Hao RJ, Du XD, Wang QH, Deng YW, Sun RJ (2019). Response to different dietary carbohydrate and protein levels of pearl oysters (*Pinctada*
*fucata*
*martensii*) as revealed by GC-TOF/MS-based metabolomics. Sci. Total Environ..

[17] Nobre AM, Robertson-Andersson D, Neori A, Sankar K (2010). Ecological–economic assessment of aquaculture options: Comparison between abalone monoculture and integrated multi-trophic aquaculture of abalone and seaweeds. Aquaculture.

[18] Vidal NP, Manzanos MJ, Goicoechea E, Guillén MD (2015). Farmed and non-selective sea bass (*Dicentrarchus*
*labrax*) volatile metabolites. A comparative study by SPME-GC/MS. J. Sci. Food Agric..

[19] Duan HB, Mao S, Xia Q, Ge HX, Liu MM, Li WQ, Feng SL, Wu XG, Dong ZG (2021). Comparisons of growth performance, gonadal development and nutritional composition among monosex and mixed-sex culture modes in the swimming crab (*Portunus*
*trituberculatus*). Aquac. Res..

[20] Deng CM, Kong LF, Yu RH, Li Q (2017). Seasonal variation in gonadal development and nutritive components in the golden shell colored strain of pacific oyster (*Crassostrea*
*gigas*). J. Fish. Sci. China.

[21] Sun Y, Zheng GD, Nissa M, Chen J, Zou SM (2020). Disruption of mstna and mstnb gene through CRISPR/Cas9 leads to elevated muscle mass in blunt snout bream (*Megalobrama*
*amblycephala*). Aquaculture.

[22] Chatchaiphan S, Thaithungchin C, Koonawootrittriron S, Na-Nakorn U (2019). Responses to mass selection in a domesticated population of snakeskin gourami, *Trichopodus** pectoralis*, Regan 1910, and confounding effects from stocking densities. Aquaculture.

[23] Wan W, Qin Y, Shi G, Li S, Liao Q, Ma H, Li J, Suo A, Ding D, Yu Z, Zhang Y (2023). Genetic improvement of aquaculture performance for tetraploid Pacific oysters, *Crassostrea*
*gigas*: A case study of four consecutive generations of selective breeding. Aquaculture.

[24] Zhang J, Li Q, Xu C, Han Z (2019). Response to selection for growth in three selected strains of the Pacific oyster *Crassostrea*
*gigas*. Aquaculture.

[25] Chen Y, Xu C, Li Q (2022). Genetic diversity in a genetically improved line of the Pacific oyster *Crassostrea*
*gigas* with orange shell based on microsatellites and mtDNA data. Aquaculture.

[26] Han Z, Li Q, Liu S, Yu H, Kong L (2019). Genetic variability of an orange-shell lineof the Pacific oyster *Crassostrea*
*gigas* during artificial selection inferred from microsatellites and mitochondrial COI sequences. Aquaculture.

[27] Hu Y, Li Q, Xu C, Liu S, Kong L, Yu H (2022). Response to selection for growth in successive mass selected generations of Iwagaki oyster *Crassostrea*
*nippona*. Aquaculture.

[28] Martinez VA (2007). Using marker data in conventional breeding programmes: A case of study in selective breeding programs of scallops. Aquaculture.

[29] Zhang ZD, Wu YP, Zhang Y, Cao Y, Chen SH, Tian Z, Li QJ, Sun XF, Chen AH (2022). Effects of adding EM bacteria and mechanical aeration on water quality, growth and antioxidant status of *Meretrix*
*meretrix* and *Exopalaemon*
*carinicauda* farmed in the clam–shrimp polyculture system. Aquac. Res..

[30] Wen X, Chen A, Wu Y, Yang Y, Xu YS, Xia WS, Zhang Y, Cao Y, Chen SH (2020). Comparative evaluation of proximate compositions and taste attributes of three Asian hard clams (*Meretrix*
*meretrix*) with different shell colors. Int. J. Food Prop..

[31] Sangster T, Major H, Plumb R, Wilson AJ, Wilson ID (2006). A pragmatic and readily implemented quality control strategy for HPLC–MS and GC–MS-based metabonomic analysis. Analyst.

[32] Want EJ, Masson P, Michopoulos F, Wilson LD, Theodoridis G, Plumb RS, Shockcor J, Loftus N, Holmes E, Nicholson JK (2012). Global metabolic profiling of animal and human tissues via UPLC-MS. Nat. Protoc..

[33] Wu YP, Chen AH, Zhang Y, Zhang ZD, Cao Y, Chen SH, Tian Z, Li QJ (2022). Metabolomics approach to assess the effect of siphonal autotomy on metabolic characteristics of razor clam *Solen*
*grandis*. Sci. Rep..

[34] Smith CA, Want EJ, O'Maille G, Abagyan R, Siuzdak G (2006). XCMS: Processing mass spectrometry data for metabolite profiling using nonlinear peak alignment, matching, and identification. Anal. Chem..

[35] Kanehisa M (2019). Toward understanding the origin and evolution of cellular organisms. Protein Sci..

[36] Karnjanapratum S, Benjakul S, Kishimura H, Tsai YH (2013). Chemical compositions and nutritional value of Asian hard clam (*Meretrix*
*lusoria*) from the coast of Andaman Sea. Food Chem..

[37] Chen DW, Su J, Liu XL, Yan DM, Lin Y, Jiang WM, Chen XH (2012). Amino acid profiles of bivalve Mollusks from Beibu Gulf, China. J. Aquat. Food Prod. Technol..

[38] Zeng X, Xia W, Yang F, Jiang Q (2013). Changes of biogenic amines in Chinese low-salt fermented fish pieces (Suan yu) inoculated with mixed starter cultures. Int. J. Food Sci. Technol..

[39] Yamaguchi S (1998). Basic properties of umami and its effects on food flavor. Food Rev. Int..

[40] Chen DW, Zhang M (2007). Non-volatile taste active compounds in the meat of Chinese mitten crab (*Eriocheir*
*sinensis*). Food Chem..

[41] Zhuang K, Wu N, Wang X, Wu X, Wang S, Long X, Wei X (2016). Effects of 3 feeding modes on the volatile and nonvolatile compounds in the edible tissues of female Chinese mitten crab (*Eriocheir*
*sinensis*). J. Food Sci..

[42] Ishiwatari N, Fukuoka M, Hamada-Sato N, Sakai N (2013). Decomposition kinetics of umami component during meat cooking. J. Food Eng..

[43] Shi QQ, Han G, Liu Y, Jiang JJ, Jia YY, Li XG (2022). Nutrient composition and quality traits of dried jujube fruits in seven producing areas based on metabolomics analysis. Food Chem..

[44] Hao R, Wang Z, Yang C, Deng Y, Zheng Z, Wang Q, Du X (2018). Metabolomic responses of juvenile pearl oyster *Pinctada*
*maxima* to different growth performances. Aquaculture.

[45] Yuan J, Karimi A, Zornes S, Goodgame S, Mussini F, Lu C, Waldroup PW (2012). Evaluation of the role of glycine in low-protein amino acid-supplemented diets. J. Appl. Poultry Res..

[46] Kube S, Gerber A, Jansen JM, Schiedek D (2006). Patterns of organic osmolytes in two marine bivalves, *Macoma*
*balthica*, and *Mytilus* spp., along their European distribution. Mar. Biol..

[47] Liu XL, Ji CL, Zhao JM, Wu HF (2013). Differential metabolic responses of clam *Ruditapes*
*philippinarum* to *Vibrio*
*anguillarum* and *Vibrio*
*splendidus* challenges. Fish Shellfish Immunol..

[48] Ji CL, Wu HF, Liu XL, Zhao JM, Yu JB, Yin XL (2013). The influence of salinity on toxicological effects of arsenic in digestive gland of clam *Ruditapes*
*philippinarum* using metabolomics. Chin. J. Oceanol. Limnol..

[49] Meng J, Zhu Q, Zhang L, Li C, Li L, She Z, Huang B, Zhang G (2013). Genome and transcriptome analyses provide insight into the euryhaline adaptation mechanism of *Crassostrea*
*gigas*. PLoS ONE.

[50] Young T, Alfaro AC, Villas-Bôas SG (2016). Metabolic profiling of mussel larvae: Effect of handling and culture conditions. Aquacult. Int..

[51] Raven WM, Marielle E, Clayton C, John T, Ten HG, Nicolaas D (2022). Increased amino acid turnover and net protein breakdown but preserved muscle and cognitive function in obese middle-age adults. Curr. Dev. Nutr..

