# Supplementary material for: Comparison of the flavor qualities between two varieties of Mercenaria mercenaria
Source: Sci Rep. 2023 Aug 11;13:13047. doi: 10.1038/s41598-023-39757-4 (PMC10421933; doi:10.1038/s41598-023-39757-4)
Supplement: Supplementary file 1 — Supplementary Table 1. [file 41598_2023_39757_MOESM1_ESM.pdf]

Table Supplementary table 1 Pathway enrichment of differential metabolites								
Pathways	Total	Count	P	-log(p)	Holm adjust	FDR	Impact	compounds
Alanine, aspartate and glutamate metabolism	28	8	6.17E-06	11.996	0.00093149	0.000469	0.75502	C00049;C00152;C03794;C00041;C00064;C00025;C00334;C12270
D-Arginine and D-ornithine metabolism	5	2	0.001008	6.9	0.14814	0.02553	0.5	C01110;C00062
Arginine and proline metabolism	66	7	0.000984	6.9236	0.14567	0.02553	0.41285	C00062;C00334;C00148;C00077;C00025;C01165;C00431
Arginine biosynthesis	23	6	0.000167	8.699	0.025013	0.008449	0.34742	C00062;C00064;C00437;C00025;C00049;C00077
Glutathione metabolism	37	5	0.012419	4.3885	1	0.20974	0.28602	C00051;C00025;C01879;C00077;C00072
D-Glutamine and D-glutamate metabolism	8	2	0.034967	3.3533	1	0.36007	0.2619	C00025;C00064
Pantothenate and CoA biosynthesis	24	3	0.013895	4.2762	1	0.2112	0.24263	C00864;C00049;C00106
Pyrimidine metabolism	67	5	0.11167	2.1922	1	0.80827	0.22494	C00105;C00064;C00055;C00214;C00106
Phenylalanine, tyrosine and tryptophan biosynthesis	32	4	0.001568	6.4579	0.22894	0.03405	0.2182	C00296;C04302;C00079;C00078
Vitamin B6 metabolism	22	3	0.049586	3.004	1	0.44336	0.19505	C00314;C00534;C00847
Tryptophan metabolism	51	1	0.33074	1.1064	1	1	0.17804	C00078
Purine metabolism	80	7	0.032052	3.4404	1	0.36007	0.17419	C00064;C00008;C00020;C03794;C00212;C00144;C00387
Phenylalanine metabolism	51	4	0.12918	2.0466	1	0.88052	0.16878	C00079;C00423;C00811;C05620
alpha-Linolenic acid metabolism	26	1	0.27644	1.2858	1	1	0.16737	C16324
Histidine metabolism	33	4	0.035534	3.3373	1	0.36007	0.15416	C00025;C00135;C00049;C05570
Cysteine and methionine metabolism	55	4	0.020584	3.8832	1	0.2703	0.15341	C02989;C00073;C00049;C00041
Isoflavonoid biosynthesis	44	1	0.82192	0.19612	1	1	0.14409	C00858
Biosynthesis of amino acids	123	17	6.99E-09	18.779	1.06E-06	1.06E-06	0.13563	C00188;C00049;C00025;C00437;C00077;C00062;C00041;C00666;C00064;C04302;C00079;C00073;C01165;C00148;C00152;C00135;C00078
Ascorbate and aldarate metabolism	41	1	0.79956	0.2237	1	1	0.12391	C00072
Glycine, serine and threonine metabolism	45	4	0.00832	4.7891	1	0.15807	0.12308	C06231;C00049;C00188;;C00078
Nicotinate and nicotinamide metabolism	50	3	0.29889	1.2077	1	1	0.11587	C00049;C00253;C00334
Steroid hormone	88	2	0.66935	0.40145	1	1	0.09584	C01227;C00951
Lysine biosynthesis	33	2	0.13903	1.9731	1	0.88052	0.09577	C00666;C00049

2-Oxocarboxylic acid metabolism	106	7	0.02134	3.8472	1	0.2703	0.09328	C00049;C00025;C00417;C00437;C00079;C00078;C00077
Peptidoglycan biosynthesis	24	1	0.075017	2.59	1	0.57013	0.07622	C00105
Biosynthesis of secondary metabolites	779	26	0.13539	1.9996	1	0.88052	0.07566	C00079;C04302;C00423;C00811;C01197;C00858;C00049;C00062;C00077;C00666;C01433;C00152;C00020;C00008;C00148;C00078;C00437;C00025;C00188;C00135;C01165;C00417;C00073;C00864;C16324;C00072
Phenylpropanoid	57	4	0.17233	1.7583	1	0.92898	0.0729	C00079;C01197;C00811;C00423
Aminobenzoate degradation	64	3	0.44587	0.80772	1	1	0.07167	C00292;C00261;C06608
Biosynthesis of antibiotics	716	16	0.9397	0.062193	1	1	0.06479	C00049;C00025;C00079;C00077;C00062;C00148;C00437;C00666;C01165;C00188;C00020;C00008;C04302;C00078;C00397;C00417
Amino sugar and nucleotide sugar metabolism	105	1	0.91926	0.084187	1	1	0.0632	C00140
Glycerophospholipid metabolism	47	3	0.26724	1.3196	1	1	0.0598	C01996;C00346;C00670
Valine, leucine and isoleucine biosynthesis	21	1	0.055476	2.8918	1	0.46847	0.05941	C00188
Phosphonate and phosphinate metabolism	36	1	0.75593	0.2798	1	1	0.05449	C03557
Metabolic pathways	1660	53	0.18396	1.693	1	0.93206	0.05251	C00072;C00025;C00152;C00049;C00188;C06231;C00073;C00408;C00148;C00077;C00062;C00135;C04302;C00079;C05588;C00078;C01165;C00437;C00334;C00423;C03794;C00020;C00008;C00212;C00144;C00387;C00064;C00055;C00105;C00106;C00051;C00041;C03557;C01227;C00951;C00314;C00253;C00864;C00811;C01197;C12448;C00214;C00417;C01433;C00666;C00346;C12270;C00140;C14772;C00534;C00847;C01110;C01571;C02679
Citrate cycle (TCA cycle)	20	1	0.54245	0.61165	1	1	0.04799	C00417
Butirosin and neomycin biosynthesis	75	2	0.56168	0.57682	1	1	0.04703	C00025;C00397
Monoterpenoid biosynthesis	32	1	0.36186	1.0165	1	1	0.04634	C01433
Taurine and hypotaurine metabolism	20	2	0.17671	1.7333	1	0.92898	0.04392	C00041;C00025
Toluene degradation	41	1	0.79956	0.2237	1	1	0.03168	C00261
Galactose metabolism	44	1	0.82192	0.19612	1	1	0.02935	C00492
Glyoxylate and dicarboxylate metabolism	52	3	0.32012	1.1391	1	1	0.02734	C00417;C00025;C00064

Arachidonic acid metabolism	35	1	0.16661	1.7921	1	0.92898	0.01997	C14772
Sphingolipid metabolism	21	1	0.56004	0.57974	1	1	0.01423	C00346
Butanoate metabolism	38	2	0.43015	0.84362	1	1	0.0121	C00334;C00025
Microbial metabolism in diverse environments	756	19	0.98053	0.019664	1	1	0.01063	C00261;C00025;C00188;C06231;C00049;C00666;C03557;C00292;C00423;C00041;C01227;C00072;C00253;C00847;C00534;C00314;C00334;C00417;C00064
Porphyrin and chlorophyll metabolism	98	2	0.73821	0.30353	1	1	0.00852	C00025;C00188
Degradation of aromatic compounds	219	3	0.99202	0.0080122	1	1	0.00696	C00261;C00423;C00811
Carbon fixation pathways in prokaryotes	44	1	0.82192	0.19612	1	1	0.00297	C00417
Carbon metabolism	111	3	0.8054	0.21641	1	1	0.00293	C00049;C00041;C00025
Lysine degradation	44	2	0.50674	0.67976	1	1	9.00E-04	C00408;C00431
Tyrosine metabolism	59	2	0.667	0.40497	1	1	0.00032	C05588;C00811
Ubiquinone and other terpenoid-quinone biosynthesis	51	2	0.58728	0.53225	1	1	0.00013	C00811;C00423
beta-Alanine metabolism	25	6	0.000275	8.2004	0.040907	0.010433	0	C00049;C00864;C05340;C00334;C00106;C00135
C5-Branched dibasic acid metabolism	19	2	0.03879	3.2496	1	0.36851	0	C00417;C00025
Carbapenem biosynthesis	26	3	0.075017	2.59	1	0.57013	0	C00025;C00148;C01165
Nitrogen metabolism	19	2	0.16292	1.8145	1	0.92898	0	C00064;C00025
D-Alanine metabolism	5	1	0.17724	1.7303	1	0.92898	0	C00041
Carbon fixation in photosynthetic organisms	22	2	0.20478	1.5858	1	1	0	C00049;C00041
Zeatin biosynthesis	24	2	0.2333	1.4554	1	1	0	C00020;C00008
Cyanoamino acid	45	3	0.24638	1.4009	1	1	0	C00049;C00152;C00079
Tropane, piperidine and pyridine alkaloid	25	2	0.24766	1.3957	1	1	0	C00079;C00408
Monobactam biosynthesis	29	2	0.30517	1.1869	1	1	0	C00062;C00049
Novobiocin biosynthesis	28	1	0.30517	1.1869	1	1	0	C00148
Clavulanic acid biosynthesis	10	1	0.32324	1.1294	1	1	0	C00062
Steroid degradation	18	1	0.50515	0.68289	1	1	0	C01227
Selenocompound metabolism	21	1	0.56004	0.57974	1	1	0	C00041
Ether lipid metabolism	21	1	0.56004	0.57974	1	1	0	C00670
Indole alkaloid biosynthesis	21	1	0.56004	0.57974	1	1	0	C00078

Fatty acid biosynthesis	49	2	0.56529	0.57042	1	1	0	C02679;C01571
Glucosinolate biosynthesis	50	2	0.57639	0.55098	1	1	0	C00079;C00078
Glycolysis / Gluconeogenesis	31	1	0.70289	0.35256	1	1	0	C06186
Pentose phosphate pathway	35	1	0.74614	0.29285	1	1	0	C00121
Folate biosynthesis	36	1	0.75593	0.2798	1	1	0	C02587
